# Association between football training experience and attentional networks in boys aged 7–11 years: a cross-sectional study

**DOI:** 10.3389/fpubh.2026.1839375

**Published:** 2026-06-17

**Authors:** Jiaxian Geng, Fanying Meng

**Affiliations:** Institute of Physical Education, Huzhou Normal University, Huzhou, China

**Keywords:** attentional network, boys, executive control, football training experience, orienting

## Abstract

**Background:**

While physical exercise has been shown to benefit the attentional networks in adolescents, older adults, and clinical populations, the specific associations regarding typically developing children remain unclear. This study aimed to investigate the association between football training experience and attentional sub-networks in boys aged 7–11 years.

**Methods:**

A total of 94 boys aged 7–11 years were recruited in this cross-sectional study and categorized into a football group (*n* = 47, ≥1 year of systematic football training) and a control group (*n* = 47, with no systematic extracurricular sports training, engaging only in regular school physical education classes). All participants completed the Attention Network Test to assess the efficiency of three sub-networks: alerting, orienting, and executive control.

**Results:**

The football group showed significantly higher efficiency in both the alerting and executive control networks compared with the control group, with the latter characterized by a smaller conflict effect. Conversely, no significant group difference was found in orienting network efficiency.

**Conclusion:**

These findings suggest a selective association between football training experience and attentional networks, specifically highlighting advantages in the alerting and executive control networks. This study provides empirical support for considering football as a developmentally appropriate activity that is associated with specific attentional advantages in boys aged 7–11 years.

## 1 Introduction

As a foundational cognitive process, attention facilitates the filtering of relevant stimuli, the maintenance of task-related engagement, and the dynamic allocation of cognitive resources (1). This mechanism serves as a critical foundation for efficient information processing, which is essential for the generation of adaptive behaviors. Modern neuroscientific perspectives define attention not as a single resource, but as a neural regulatory system organized into three distinct, neuroanatomically independent sub-networks: alerting, orienting, and executive control (1). The alerting network involves the ability to achieve and maintain sensitivity to incoming stimuli (2), and is localized within the brainstem reticular system, thalamus, and frontal/parietal cortices (3). The orienting network refers to the capacity to shift attentional resources toward targets based on cues (4), involving the frontal eye fields, the intraparietal sulcus/superior parietal lobe, the temporoparietal junction and the ventral frontal cortex (3). Finally, the executive control network monitors and resolves environmental conflicts by inhibiting irrelevant information to ensure goal attainment (5), a process mediated by the prefrontal cortex (dorsolateral prefrontal cortex, inferior frontal gyrus) and the anterior cingulate cortex. Utilizing this framework, Fan et al. developed the Attentional Network Test (ANT) to quantify these functions (6). To date, researchers have adopted the ANT paradigm to investigate attentional network functions across diverse populations, including patients (7), athletes (8), as well as older and younger adults (9, 10). This widespread use demonstrates the paradigm’s high validity and broad applicability.

Research indicated that human attention network is highly plastic, with its functional efficiency optimized through targeted interventions such as cognitive training (11), meditation (12), and physical exercise (13). Physical exercise is a cost-effective means to selectively enhance attentional sub-networks. These benefits follow a hierarchy, ranging from transient acute effects to more enduring long-term adaptations. Specifically, a single exercise bout temporarily enhances alerting (14) or executive control (15). Whereas long-term regular exercise produces more stable neurocognitive optimization. The magnitude of these benefits depends on sport modality, open-skill versus closed-skill (16). Open-skill sports (e.g., football, sparring) are played in dynamic, unpredictable, and externally-paced environments that require constant adjustment to changing stimuli. Conversely, closed-skill sports (e.g., swimming, running) are played in stable, predictable, and self-paced settings (16, 17). While open-skill sports are hypothesized to yield greater cognitive gains due to their higher cognitive demand (8, 10), the empirical evidence is mixed, with outcomes frequently moderated by participant demographics, training protocols, and the cognitive domains examined. For example, martial arts practitioners with over 2 years of experience have shown higher alerting efficiency compared to control groups (18). Although such findings from adults samples may not directly generalize to children, they nevertheless provide a critical theoretical foundation for investigating pre-adolescent children (e.g., ages 7–11). This age period represents a key developmental window characterized by heightened neural plasticity and the rapid maturation of attentional systems.

In concrete terms, during middle childhood, the prefrontal and parietal cortices undergo significant structural and functional development, laying the groundwork for the maturation of attentional systems. These advancements are pivotal for children’s cognitive (19), academic (20), and social trajectories (21). Despite this developmental potential, childhood attention is increasingly compromised by modern environmental factors, such as electronic device overuse (22), high academic pressure (23, 24), intrusive parenting (25), and poor sleep (26, 27). In this context, physical exercise represents a modifiable lifestyle factor that may relate to attentional function in children. Although the relationship between physical exercise and attention has been extensively studied, existing research has predominantly concentrated on college students and older adults, creating a critical research gap regarding attentional networks in pediatric populations. In particular, whether football training experience is associated with the three ANT-based attentional networks (alerting, orienting, and executive control) in children aged 7–11 remains largely unexplored. To address this gap, the current study utilized the ANT paradigm to compare attentional network efficiency in children aged 7–11 with and without football training experience. We hypothesized that football exercise would be associated with enhanced efficiency in the alerting and executive control networks, given the cognitive demands of football, which require sustained alertness to dynamic environmental changes and rapid inhibition of prepotent responses. The orienting network was examined as an exploratory outcome, as previous studies have generally failed to find exercise-induced improvements in orienting (14, 16).

## 2 Materials and methods

### 2.1 Participants

Sample size was determined using G*Power 3.1 software (28). Based on the effect sizes reported in a recent meta-analysis of football training on children’s cognitive performance (Hedges’ *g* = 0.77 for attention) (29), we adopted a conservative effect size of Cohen’s *d* = 0.60 to account for potential sample heterogeneity and attrition. For an independent-samples *t*-test with *α* = 0.05 and power = 0.80, the power analysis indicated that a minimum of 90 participants (45 per group) was required. Our final sample (*N* = 94, 47 per group) exceeds this requirement.

To minimize environmental and academic confounders, a total of 94 boys aged 7–11 years were recruited from three primary schools within the same educational group and administrative district of Huzhou City. These schools share identical curricula, synchronized assessments, and comparable socioeconomic backgrounds. The study focused exclusively on male participants to minimize sex-related physiological and developmental heterogeneity. Furthermore, too few female students had at least 1 year of systematic football training to form a separate group. Participants were categorized into either a football group or a control group based on their training history. Both groups attended the standard, mandatory school physical education (PE) classes as part of their regular school curriculum. In addition to these routine PE activities, the football group (*n* = 47) had an average of 2.59 years (SEM = 0.12) of systematic football training. The regular training schedule comprised 3–5 sessions per week (90 min per session) and a weekend match, totaling a minimum of 4.5 h of organized football weekly. Assuming approximately 40 training weeks per year, the estimated annual cumulative training duration ranged from 180 to 300 h. These sessions included technical drills (e.g., passing, dribbling, shooting), small-sided games, tactical exercises and along with physical conditioning and warm-ups. In contrast, the control group (*n* = 47) engaged strictly and exclusively in these standard school PE activities and had no history of any systematic, extracurricular sports training. Inclusion criteria for all participants were: right-handed, normal or corrected-to-normal visual acuity, and no history of major physical or mental disorders. Exclusion criteria included a formal diagnosis of learning disabilities, neurodevelopmental disorders (including ADHD), or parent-reported attentional problems.

Prior to the experiment, the purpose and procedures were fully explained to the guardians of all participants, and written informed consent was obtained from each of them. It was emphasized that participation was voluntary. Upon completion of the experiment, each participant received a small gift as compensation. This research was approved by the Ethics Committee of Huzhou Normal University (No. 202406–02) and adhered to the Declaration of Helsinki and relevant ethical guidelines for behavioral research.

### 2.2 ANT task procedure

**Figure 1:**
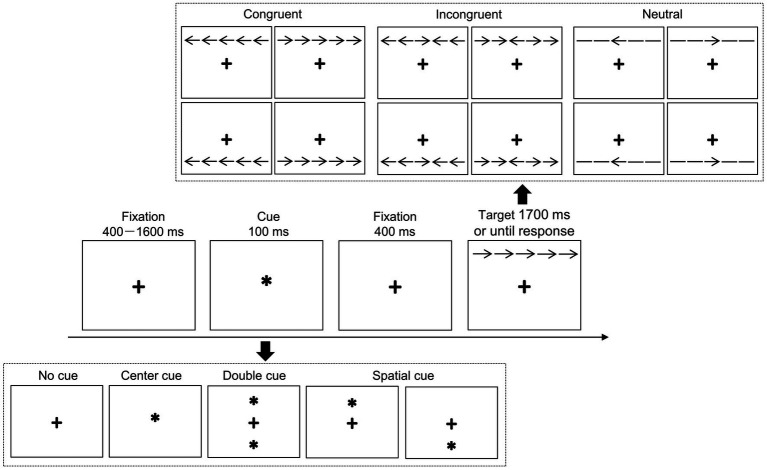
Sequence of events within a single trial of the attention network test.

Based on the methodological framework established by Chang et al. (15), the ANT was programmed using E-Prime 3.0 software (Psychology Software Tools, Pittsburgh, PA, USA). Participants were seated 60 cm from a 17-inch Lenovo monitor, where stimuli were presented against a neutral gray background to ensure clarity and minimize visual interference. The trial sequence and event timings of the ANT are illustrated in Figure 1.

Each trial began with a fixation cross presented for a jittered duration (400–1,600 ms) to mitigate anticipatory effects. A cue was then presented for 100 ms, consisting of one of four conditions: (a) no cue (the fixation cross remained unchanged); (b) center cue (the fixation cross was replaced by a single asterisk); (c) double cue (asterisks appeared simultaneously above and below the fixation cross); or (d) spatial cue (a single asterisk appeared randomly above or below the fixation cross). Following the cue, the fixation cross was re-presented for 400 ms. Subsequently, a response stimulus appeared either above or below the fixation cross, consisting of a central target arrow flanked by four arrows (two on each side). Three flanker types were implemented based on the relationship between the target and flankers: (a) congruent (target and flankers oriented in the same direction, e.g., ← ← ← ← ←); (b) incongruent (target and flankers oriented in opposite directions, e.g., → → ← → →); or (c) neutral condition (the target was an arrow, while flankers were non-directional horizontal lines, e.g., −- → --). Participants were instructed to respond as quickly and accurately as possible to the direction of the central target by pressing the corresponding key on a numeric keypad: the “1” key for a left pointing target (pressed with the left index finger) and the “3” key for a right pointing target (pressed with the right index finger). The response stimulus remained on screen until a response was made or until the 1700 ms timeout was reached. Trials without a valid response within this period were coded as “misses” and proceeded directly to the next trial.

Prior to the formal experiment, participants completed a 36-trial practice block to ensure full familiarity with the task. All 12 combinations of cue types (4) and flanker conditions (3) were presented in a randomized sequence during this phase, with immediate feedback regarding their reaction time and response accuracy. The formal experiment comprised192 trials, divided into four blocks of 48 trials each, with an equal distribution of trials across all 12 experimental conditions. No feedback was provided during the formal experiment.

### 2.3 Statistical analysis

#### 2.3.1 Demographic characteristics

To assess baseline equivalence between the two groups, independent samples *t*-tests were conducted on key demographic, including age, height, weight, and body mass index (BMI).

#### 2.3.2 Reaction times and accuracy rates

To eliminate potential noise caused by performance errors and statistical outliers, we first excluded miss trials (3.38%), trials with incorrect responses (3.9%) and those with reaction times (RTs) outside ± 3 standard deviations from the mean (0.44%) for the RTs analysis. Accuracy rates were calculated as the percentage of correct responses relative to the total number of valid trials (i.e., the sum of correct and incorrect responses) after excluding miss trials and RT outliers. Subsequently, a three-way mixed-design analysis of covariance (ANCOVA) was conducted separately for the mean RTs and accuracy rates, with age included as a covariate. The within-subject factors were cue type (no cue, center cue, double cue, spatial cue) and flanker type (congruent, incongruent, neutral), while the between-subject factor was group (football group, control group).

#### 2.3.3 Attentional networks

Statistical analyses of attentional networks were performed separately for the alerting, orienting, and executive control. For each network, a two-way mixed-design analysis of covariance (ANCOVA) was conducted on the mean RTs, with age included as a covariate. The within-subjects factor varied across the three analyses: cue type (no cue, double cue) for the alerting network; cue type (center cue, spatial cue) for the orienting network; and flanker type (congruent, incongruent) for the executive control network. For all analyses, the between-subjects factor was group (football group, control group).

The efficiency of each attentional network was calculated based on mean RTs for correct trials, defined as follows: (a) the alerting network effect was calculated by subtracting the double cue RT from the no cue RT; (b) the orienting network effect was calculated by subtracting the spatial cue RT from the center cue RT; and (c) the executive control network effect was calculated by subtracting the congruent RT from the incongruent RT (6). To examine group differences in each attentional network, independent samples *t*-tests were performed on the efficiency of the alerting, orienting, and executive control networks.

All statistical analyses were performed using SPSS Statistics 26.0. The significance level was set at *α* = 0.05 for two-tailed tests. For all mixed-design and repeated-measures ANCOVAs, Greenhouse–Geisser corrections were applied to the degrees of freedom and *p*-values when Mauchly’s test indicated a violation of the sphericity assumption. Significant interactions were followed up with Bonferroni-adjusted *post hoc* comparisons.

## 3 Results

### 3.1 Demographic characteristics

Independent samples *t*-tests revealed no significant differences between the football group and the control group in age (t(92) = 1.40, *p* = 0.22), height (t(92) = −0.78, *p* = 0.44), weight (t(92) = −0.56, *p* = 0.58), or body mass index (t(92) = 0.09, *p* = 0.93) (all *ps* > 0.05). These results confirm that the groups were homogeneous on these baseline measures (Table 1).

**Table 1:** Demographic and physical characteristics of the football and control groups (mean ± standard error).

Characteristic	Football group	Control group
n	47	47
Age (years)	9.11 ± 0.18	8.79 ± 0.19
Height (cm)	139.56 ± 1.67	140.99 ± 1.42
Body mass (kg)	38.85 ± 1.13	40.09 ± 1.88
Body mass index (kg/m^2^)	19.78 ± 0.30	19.72 ± 0.59

### 3.2 Reaction times

All reported degrees of freedom for within-subjects factors are Greenhouse–Geisser corrected. A 4 × 3 × 2 mixed ANCOVA on RTs (with age as a covariate) revealed statistically significant main effects of cue type (*F*(3, 89) = 3.85, *p* = 0.02, $\eta_{p}^{2}$ = 0.04) and flanker type (*F*(2, 90) = 18.30, *p* = 0.00, $\eta_{p}^{2}$ = 0.17).

The interaction between cue type and flanker type was also statistically significant (*F*(6, 86) = 2.66, *p* = 0.02, $\eta_{p}^{2}$ = 0.03). Bonferroni-adjusted *post hoc* comparisons revealed a consistent pattern across all cue conditions: participants responded significantly faster in both congruent and neutral conditions compared to incongruent condition (all *ps* < 0.001). However, the relationship between neutral and congruent conditions shifted depending on the cue: participants responded significantly faster in neutral condition than congruent condition under no cue and double cue conditions (all *ps* < 0.05), while this difference disappeared under center cue and spatial cue conditions (all *ps* > 0.05). Across all flanker types, the RTs were shortest observed in the spatial cue condition and longest in the no cue condition (all *ps* < 0.05). RTs in the center cue and double cue conditions did not differ significantly (*p* > 0.05).

The interaction between flanker type and group was also statistically significant (*F*(2, 90) = 3.83, *p* = 0.03, $\eta_{p}^{2}$ = 0.04). Simple effects analysis revealed a distinct pattern between groups. In the football group, RTs in the neutral condition were significantly shorter than those in both the congruent and incongruent conditions (all *ps* < 0.01). Furthermore, RTs in the congruent condition were significantly shorter than those in the incongruent condition (*p* < 0.001). In the control group, RTs in both the congruent and neutral conditions were significantly shorter than those in the incongruent condition (all *ps* < 0.001), but no significant difference was found between the congruent and neutral conditions (*p* > 0.05).

The main effect of group was not statistically significant (*F*(1, 91) = 0.30, *p* = 0.59, $\eta_{p}^{2}$ = 0.00), nor did the two-way interaction between cue type and group reach statistical significance. (*F*(3, 89) = 1.85, *p* = 0.15, $\eta_{p}^{2}$ = 0.02). Similarly, the three-way interaction among cue type, flanker type and group was non-significant (*F*(6, 86) = 1.92, *p* = 0.08, $\eta_{p}^{2}$ = 0.02) (Table 2).

**Table 2:** Mean reaction times for correct trials in the attention network test for the football and control groups (mean ± standard error).

Group	Cue type	Flanker type		
		Congruent	Incongruent	Neural
Football	No cue	661.85 ± 15.50	742.86 ± 16.02	650.72 ± 13.69
	Center cue	620.59 ± 15.86	726.31 ± 15.68	615.71 ± 14.99
	Double cue	620.36 ± 14.47	722.26 ± 14.25	601.67 ± 13.74
	Spatial cue	586.60 ± 17.19	664.43 ± 16.50	577.85 ± 16.93
Control	No cue	674.15 ± 14.69	766.60 ± 18.34	657.99 ± 15.99
	Center cue	636.45 ± 15.86	749.74 ± 15.22	625.35 ± 16.03
	Double cue	636.34 ± 16.71	758.14 ± 17.68	636.13 ± 16.85
	Spatial cue	590.02 ± 14.80	707.84 ± 19.03	597.34 ± 15.42

### 3.3 Accuracy rates

A parallel analysis on accuracy revealed no significant main effects or interactions involving cue type, flanker type, or group (all *ps* < 0.001) (Table 3).

**Table 3:** Mean accuracy rates in the attention network test for the football and control groups (mean ± standard error).

Group	Cue type	Flanker type		
		Congruent	Incongruent	Neural
Football	No cue	99.09 ± 0.46	93.57 ± 1.24	97.28 ± 0.75
	Center cue	97.83 ± 0.56	89.89 ± 1.24	97.83 ± 0.56
	Double cue	97.81 ± 0.63	92.28 ± 1.29	98.19 ± 0.56
	Spatial cue	98.60 ± 0.42	93.72 ± 1.15	97.81 ± 0.60
Control	No cue	98.32 ± 0.74	92.15 ± 1.44	96.36 ± 1.10
	Center cue	98.98 ± 0.42	91.45 ± 1.41	97.28 ± 0.79
	Double cue	98.70 ± 0.70	91.64 ± 1.48	97.79 ± 1.25
	Spatial cue	98.98 ± 0.42	94.40 ± 1.28	97.94 ± 0.78

### 3.4 Attentional networks

#### 3.4.1 Alerting network

**Figure 2:**
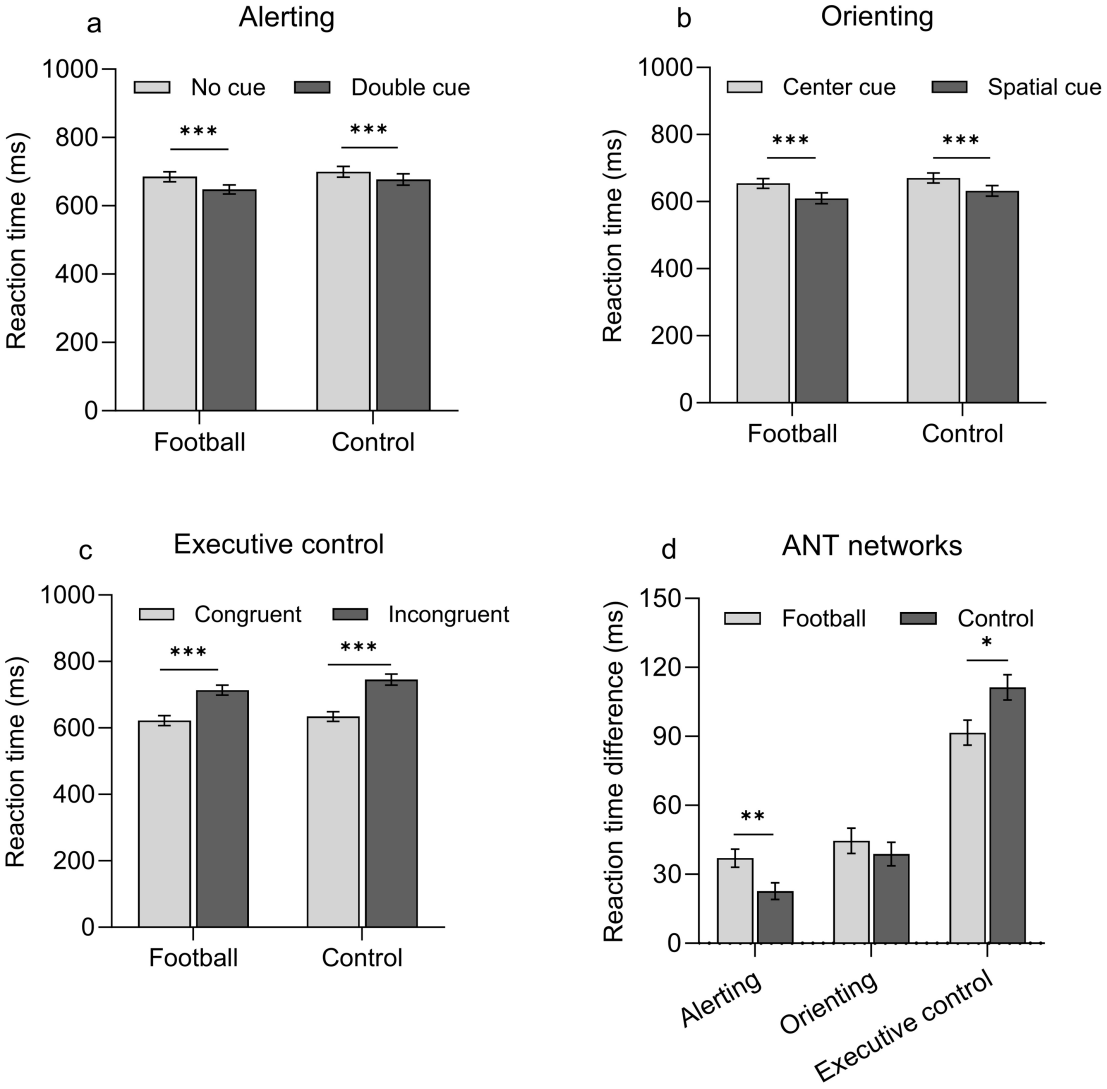
Mean reaction times (ms) across attentional networks for football and control groups. **(a)** Alerting network: the network efficiency was calculated by subtracting the double cue RT (dark gray bars) from the no cue RT (light gray bars). **(b)** Orienting network: the network efficiency was calculated by subtracting the spatial cue RT (dark gray bars) from the center cue RT (light gray bars). **(c)** Executive control network: the network efficiency was calculated by subtracting the congruent RT (light gray bars) from the incongruent RT (dark gray bars). **(d)** Network efficiencies: a comprehensive comparison of the three attentional network efficiencies between the football group (light gray bars) and the control group (dark gray bars). Error bars represent the standard error of the mean (SEM). Statistical significance between groups is indicated by asterisks (*p < 0.05, **p < 0.01, ***p < 0.001).

The 2 × 2 ANCOVA with age as a covariate revealed a significant main effect of cue type (*F*(1, 91) = 8.22, *p* = 0.01, $\eta_{p}^{2}$ = 0.08). The interaction between cue type and group was also statistically significant (F(1, 91) = 8.21, *p* = 0.01, $\eta_{p}^{2}$ = 0.08). *Post hoc* comparisons with Bonferroni correction revealed no significant group differences in RTs for either cue condition (both *ps* > 0.05). Conversely, participants in both groups benefited from cueing, showing significantly shorter RTs in the double cue compared to the no cue condition (both *ps* < 0.001). The main effect of group was not significant (*F*(1, 91) = 0.36, *p* = 0.55, $\eta_{p}^{2}$ = 0.00) (Figure 2a).

An independent samples *t*-test revealed that the football group (mean ± standard error: 37.04 ± 3.93) demonstrated significantly greater alerting network efficiency than the control group (22.71 ± 3.60) (t(92) = 2.69, *p* = 0.01, Cohen’s *d* = 0.56, 95% CI for mean difference [3.75, 24.92]) (Figure 2d; Table 4).

**Table 4:** Summary of the results for the three attentional networks.

Attentional networks	Factor	ANCOVA/*t*-test
Alerting	Cue type (no/double)	F(1, 91) = 8.22, *p* = 0.01, $\eta_{p}^{2}$ = 0.08
	Group (football/control)	*F*(1, 91) = 0.36, *p* = 0.55, $\eta_{p}^{2}$ = 0.00
	Cue type × Group	F(1, 91) = 8.21, *p* = 0.01, $\eta_{p}^{2}$ = 0.08
	RT(no cue) – RT(double cue)	t(92) = 2.69, *p* = 0.01, Cohen’s *d* = 0.56
Orienting	Cue type (central/spatial)	F(1, 91) = 1.75, *p* = 0.19, $\eta_{p}^{2}$ = 0.02
	Group (football/control)	F(1, 91) = 0.23, *p* = 0.63, $\eta_{p}^{2}$ = 0.00
	Cue type × Group	F(1, 91) = 0.54, *p* = 0.47, $\eta_{p}^{2}$ = 0.01
	RT(central cue) – RT(spatial cue)	t(92) = 0.78, *p* = 0.44, Cohen’s *d* = 0.16
Executive control	Flanker type (congruent/ incongruent)	F(1, 91) = 19.72, *p* = 0.00, $\eta_{p}^{2}$ = 0.18
	Group (football/control)	F(1, 91) = 0.35, *p* = 0.56, $\eta_{p}^{2}$ = 0.00
	Flanker type × Group	F(1, 91) = 5.77, *p* = 0.02, $\eta_{p}^{2}$ = 0.06
	RT(incongruent) – RT(congruent)	t(92) = −2.55, *p* = 0.01, Cohen’s *d* = 0.53

#### 3.4.2 Orienting network

The results of 2 × 2 ANCOVA revealed no main effect of cue type or group, nor was there a significant interaction between them (all *ps* > 0.05) (Figure 2b).

An independent samples t-test indicated no significant difference in orienting network efficiency between the football (44.57 ± 5.45) and control groups (38.78 ± 5.10) (t(92) = 0.78, *p* = 0.44, Cohen’s *d* = 0.16, 95% CI for mean difference [−9.04, 20.62]) (Figure 2d; Table 4).

#### 3.4.3 Executive control network

The results of 2 × 2 ANCOVA revealed a significant main effect of flanker type (*F*(1, 91) = 19.72, *p* = 0.00, $\eta_{p}^{2}$ = 0.18). The interaction between flanker type and group interaction was also significant (F(1, 91) = 5.77, *p* = 0.02, $\eta_{p}^{2}$ = 0.06). Simple effects analysis indicated that no significant group differences in RTs for all flanker types (both *ps* > 0.05). Regardless of group, participants exhibited significantly shorter RTs in the congruent condition relative to the incongruent condition (both *ps* < 0.001). The main effect of group was not significant (F(1, 91) = 0.35, *p* = 0.56, $\eta_{p}^{2}$ = 0.00) (Figure 2c).

An independent samples t-test indicated that the football group (91.61 ± 5.41) exhibited significantly smaller conflict effect than the control group (111.34 ± 5.53) (t(92) = −2.55, *p* = 0.01, Cohen’s *d* = 0.53, 95% CI for mean difference [−35.07, −4.37]) (Figure 2d; Table 4).

## 4 Discussion

The present research investigated the associations between football training experience and the attentional networks (alerting, orienting, and executive control) in boys aged 7–11 years. To this end, participants were recruited from primary school grades 2 to 5 and categorized into two groups: a football group, comprising boys who had undergone at least 1 year of consistent football training, and a control group, comprising boys with no history of systematic sports training. Attentional performance was quantified via the ANT. While previous research has explored the cognitive benefits of general physical exercise, the present study is among the first to examine the associations between football training experience and attentional networks in a pediatric population. Our primary findings indicate that the football group exhibited significantly greater efficiency in the alerting and executive control networks compared to the control group, whereas no significant differences were observed regarding the orienting network efficiency. These results suggest that football training experience may be selectively associated with specific attentional sub-networks, implying that the complex, high-interference demands of football primarily relate to preparatory arousal and conflict resolution rather than spatial shifting.

### 4.1 Football exercise and alerting network

Our results indicated that both the football and control groups exhibited significantly shorter RTs in the double cue condition relative to the no cue condition. These findings align with Posner and Petersen’s Attention Network Theory (30) and subsequent empirical research (9, 10), which posit that cues accelerate response speed by rapidly activating the alerting network, particularly the phasic alertness pathway (31, 32). This activation effectively reduces the time required for information processing preparation, thereby supporting overall task efficiency. Although no significant RTs difference was found between the two groups under either the no cue or the double cue condition, a significant between-group difference emerged in alerting network efficiency. These findings tentatively suggest a potential divergence in attentional regulation strategies between the two groups. In the no cue condition, the football group exhibited higher efficiency, which might reflect a more optimal endogenous alerting strategy associated with football training experience. Although not directly quantified in the present study, such a strategy remains a speculative hypothesis that is theoretically linked to greater efficiency in cognitive resource allocation, which in turn could potentially support sustained monitoring stability. Under the double cue condition, the exogenous cues might have alleviated the cognitive demand of sustained endogenous monitoring in the football group. This proposed synergistic mechanism of endogenous preparation and exogenous guidance could support more efficient information processing. These findings align with and extend our understanding of how physical exercise relates to the alerting network. While a single session of moderate-intensity activity can transiently improve alerting network efficiency (33), consistent, long-term training appears to be associated with more profound, sustained behavioral advantages, as systematically underscored by robust meta-analytic evidence on sport expertise (13, 34).

As a prototypical open-skill sport, football is characterized by rapid transitions between attack and defence within a fluid spatial context. These demands are thought to impose a significant cognitive load on the alerting network. Thus, during training and competitive play, young players must continuously integrate multidimensional stimuli, including the positioning of teammates, tactical shifts by opponents, and transitions in ball possession (35). This high-demand environment is hypothesized to require the alerting system operate with greater stability and efficiency to maintain optimal performance. Crucially, empirical support from related team-sport research indicates that individuals with extensive strategic sports training exhibit superior attentional alerting efficiency (36). Within this context, the scenario-based demands of football might be associated with a specialized optimization of the alerting network. Specifically, football training experience may be linked to superior endogenous alertness, improving the capacity to sustain arousal, identify key stimuli, and suppress distractions.

Although neural activity was not directly recorded in the present study, our behavioral findings are conceptually consistent with established neuroscientific evidence indicating that athletes in open-skill sports like football demonstrate heightened preparatory response readiness and superior anticipatory alertness compared to non-athletes (37–39). Collectively, while direct mechanistic evidence remains to be established by future functional neuroimaging research, our behavioral findings are consistent with the view that football training experience is associated with a functional optimization of the alerting network in boys during middle childhood.

### 4.2 Football exercise and orienting network

Our results indicated that both groups responded significantly faster and more accurately in the spatial cue condition relative to the central cue condition, consistent with the well-documented spatial cueing effect (30). Valid cues orient individuals’ attention to the potential location of the upcoming target, thereby shortening attentional search and localization stages and facilitating faster responses. Invalid cues, however, draw attention to irrelevant locations, requiring attentional disengagement and shifting, which results in delayed responses, a phenomenon known as the classic cueing effect. Notably, although the orienting network efficiency of the football group was numerically higher than that of the control group, the inter-group difference did not reach statistical significance. This result is consistent with prior studies (8, 9, 16), which have also reported a selective pattern of cognitive benefits. While football training experience appears to be associated with the alerting and executive control networks, its influence on the orienting network appears comparatively weaker in existing literature (10, 13, 15).

Regarding the non-significant group differences in our study, several factors warrant discussion as theoretical possibilities, although these interpretations remain speculative in the absence of direct evidence. First, the sensitivity of the task might be a factor (36). The version of the ANT employed in the present study utilizes a fixed Stimulus-Onset Asynchrony (SOA), which might have created a ceiling effect, potentially limiting the task’s ability to challenge the orienting capacity of the participants (8). Future studies could address this by increasing task demands, such as introducing variable SOAs or manipulating cue reliability, as task difficulty may be a key moderator of exercise-related cognitive effects. Second, the specific training dose might be a consideration (40). While participants in the football group had at least 1 year of football training experience, exercise-induced cognitive adaptations often follow a complex dose–response relationship involving cumulative training load (i.e., intensity, frequency, and duration). It is plausible that the orienting network requires a higher threshold of targeted cognitive load, potentially achieved through more extended training duration or specific training intensities, to manifest behavioral changes than the alerting or executive networks (41). Given that we treated the orienting network as an exploratory component, these findings primarily suggest that a link may exist between football training experience and differential plasticity across attentional sub-networks. This hypothesis should be systematically investigated in future longitudinal research that incorporates comprehensive measures of training dose.

### 4.3 Football exercise and executive control network

To determine whether the faster RTs in the football group reflected a speed-accuracy trade-off, we analyzed the corresponding accuracy data. The overall accuracy analysis revealed no significant main effects or interactions involving group. Critically, the significant interaction between Group and Flanker Type found for RTs was not present for accuracy. This pattern, faster RTs without any accuracy cost, rules out a speed-accuracy trade-off as an explanation for the observed group differences in executive control.

The present study showed that young players in the football group exhibited significantly higher executive control efficiency compared to their counterparts in the control group. This advantage was reflected in their superior interference suppression, namely a more refined ability to inhibit impulsive responses and resolve conflict when processing conflicting stimuli. These findings align with previous research on college students and older adults (8, 10), supporting the hypothesis that open-skill sports are associated with a more efficient executive control network. By extending these results to a younger demographic, our study suggests that the executive control network benefits of open-skill exercise are not age-restricted but represent a cross-age phenomenon. Most importantly, our findings imply that football training experience may be linked to the development of the executive control network during a pivotal neurodevelopmental window, potentially contributing to the trajectory of cognitive maturation in childhood.

Accumulating evidence underscores physical exercise as an effective intervention for enhancing executive control during childhood (42, 43). Meta-analyses have demonstrated that physical exercise significantly improves cognitive function, with particularly robust effects executive control (44). This broad benefit is further elucidated by intervention studies indicating that structured ball-sport programs, typically involving sessions of at least 51 min, delivered twice weekly over 17 weeks or more, can yield significant gains in children’s executive control (45, 46). Beyond behavioral outcomes, neurophysiological evidence suggests that physical exercise modulates activation and enhances functional connectivity among key brain regions underlying cognitive control, including the prefrontal cortex (notably the dorsolateral and ventrolateral subdivisions), anterior cingulate cortex, parietal cortex, ventral striatum, motor cortex, and cerebellum (47, 48). Collectively, these regions constitute the core architecture of the executive control network, suggesting that exercise-induced neuroadaptive changes in their activation and connectivity represent a fundamental physiological mechanism underlying the enhancement of executive control.

In recent years, football has been systematically integrated into primary and secondary schools across China, benefiting from both high student engagement and robust institutional support. Beyond its fundamental health benefits, football is increasingly recognized as a critical vehicle for neurocognitive development during childhood, primarily because its impact on cognitive function is contingent on the high cognitive demands of the sport (17, 49). Unlike closed-skill sports, open-skill sports like football place players in a fast-changing environment that requires constant situational monitoring and rapid decision-making (50). According to the Cognitive Stimulation Hypothesis (51) and the Adaptation Model (52), the environmental demands of an activity are the primary regulators of executive function. What distinguishes these frameworks from others is their emphasis on cognitive engagement as the critical ingredient for neurocognitive adaptation, positing that exercise imposing high cognitive demands is more effective at inducing brain changes than low-cognitive activities (53, 54). Engaging in football exercise places young players in a fast-paced, unpredictable environment where they must constantly process complex information. This open-skill activity is thought to systematically challenge and refine executive control functions, such as filtering irrelevant distractions, resolving conflicts, and making effective decisions. Consequently, long-term participation may act as a potential mechanism for optimizing the executive control network.

Several limitations of this study warrant consideration. First, the cross-sectional design precludes us from establishing causal relationships. While our data reveal associations between football training experience and attentional networks, we cannot exclude the possibility of self-selection bias or pre-existing group differences. To clarify this relationship, future research should utilize longitudinal designs to track changes following the onset of training. Second, several key background variables remain uncontrolled at the individual level, including socioeconomic status, parental education, academic pressure, sleep patterns, and screen time. These factors can affect attentional development and may also relate to children’s sports participation. Given that these variables were not measured in the current study, we cannot rule out their confounding influences. We acknowledge this limitation and recommend that future studies systematically collect and control for these variables. Third, the study lacked precise monitoring and reporting of training intensity. Without intensity data, we were unable to fully describe the physical exertion during football training. While we provided detailed information on training frequency, session duration, and content, these parameters describe training volume rather than intensity. This limitation is inherent to our retrospective, real-world design. Future research should include objective training metrics to better characterize training attributes, including intensity indicators (e.g., wearable heart rate monitors, RPE scales), adherence data (e.g., actual attendance logs), and competition profiles (e.g., detailed competition histories). Fourth, daily physical activity levels were not measured in either group. While the control group had no systematic extracurricular sports training, we lack objective data on both groups’ daily movements. Consequently, we cannot rule out the possibility that group differences in general physical activity, rather than football training itself, partially contributed to the observed attentional advantages. To address this limitation, future studies should include three groups: an open-skill sport group (e.g., football), a closed-skill sport group (e.g., swimming), and a passive control group. Device-based activity tracking should be used to measure and balance total physical activity across groups. This design would help distinguish the attentional effects specific to open-skill sports from those of general physical activity. Finally, our findings was restricted to male participants, which limits the generalizability of these findings. Incorporating both boys and girls in future studies will be essential to determine whether these benefits are consistent across sexes.

## 5 Conclusion

This study investigated the association between football training experience and attentional networks in boys aged 7–11 years. Compared to the control group, boys with over 1 year of football training showed significantly greater efficiency in both the alerting and executive control networks. However, no significant difference was found between groups in orienting network efficiency. These findings suggest a selective association of football training experience with attentional networks, specifically highlighting advantages in the alerting and executive control networks, but not the orienting network. This study provides empirical support for considering football as a developmentally appropriate activity that is associated with specific attentional advantages in boys aged 7–11 years. Due to the cross-sectional design, causal inferences cannot be drawn from the present results.

## Data Availability

The datasets presented in this study can be found in online repositories. The names of the repository/repositories and accession number(s) can be found in the article/Supplementary material.
